# Effects of meteorological conditions on brood care in cooperatively breeding carrion crow and consequences on reproductive success

**DOI:** 10.1186/s12983-023-00504-0

**Published:** 2023-07-24

**Authors:** Eva Trapote, Daniela Canestrari, Vittorio Baglione

**Affiliations:** Departamento de Biodiversidad y Gestión Ambiental, Universidad de León, Campus de Vegazana s/n, 24071 León, Spain

**Keywords:** Cooperative breeding, Brood care, Reproductive success, Meteorological conditions, Climate change

## Abstract

Meteorological stressors (e.g., temperature and rain shortage) constrain brood provisioning in some bird species, but the consequences on reproductive success have been rarely quantified. Here we show, in a cooperatively breeding population of carrion crow *Corvus corone* in Spain, that individual feeding rates decreased significantly with rising air temperatures both in breeders and helpers, while lack of rain was associated with a significant reduction in the effort of the male helpers as compared to the other social categories. Group coordination, measured as the degree of alternation of nest visits by carers, was also negatively affected by rising temperature. Furthermore, we found that the body condition of the nestlings worsened when temperatures were high during the rearing period. Interestingly, the analysis of a long-term data set on crow reproduction showed that nestling body condition steadily deteriorated over the last 26-years. Although many factors may concur in causing population changes, our data suggest a possible causal link between global warming, brood caring behaviour and the decline of carrion crow population in the Mediterranean climatic region of Spain.

**Supplementary Information:**

The online version contains supplementary material available at 10.1186/s12983-023-00504-0.

## Introduction

Understanding how organisms respond to climate change [58, 59] is a central topic in animal ecology[15, 66, 95]. Phenological adjustments, like advanced laying dates, are common in birds [40, 65] with effects that ranged between negative [52, 56], when a mismatch arises between the peak of food availability and the energetic requirements of the developing young [93], neutral [41] or even positive [18, 53], because of increased chances to renest [51, 55]. Birds can also modulate their behaviour to cope with high temperatures, by resting in sheltered places [39, 42], or by increasing panting, wing spreading or gular fluttering to regulate the body temperature [86, 88]. Heat avoidance might trade off with other behaviours that are important for survival or reproduction [33], with possible consequences on individual fitness [34, 80, 91]. Understanding the effects of global warming on key-life behaviours, such as young provisioning, is therefore paramount, but information is still limited [69], especially in cooperatively breeding bird species, where subordinated individuals help raising young that are not their own [17, 30, 38]. In these species, reproductive success depend on the collective effort of several individuals that typically pursue different benefits (direct *vs* indirect, [29, 44, 72]), face different trade-offs between current and future reproduction [28, 46, 47, 87] and therefore vary largely in their investment in brood provisioning [3, 67]. The effect of external stressors, like increasing temperatures and rain shortage, may therefore affect group members in different ways, with complex consequences on the dynamic of cooperation that urgently need to be addressed.

One reason behind our incomplete understanding of brood care in birds is that most research, particularly on cooperative species, has focused so far on quantifying the individual contribution of carers, overlooking other important dimensions of this behaviour [82]. Recent studies have uncovered that reproductive success can depend on the coordination of nest visits among different group members. In long tailed tits *Aegithalos caudatus*, for example, joint visits at the nest (usually referred to as “synchrony” in the literature, e.g. [61] reduce the risk of brood predation [11], whereas, in carrion crows *Corvus corone*, alternation of carers in provisioning the brood (also referred to as “turn taking”, e.g. [81] improves the body condition of the young and their post-fledging survival [90]. Therefore, to fully understand how meteorological stressors affect the provisioning behaviour of birds and what are the consequences on reproduction, a comprehensive approach shall be adopted.

Some short-term studies have shown that weather conditions affect nest visitation rate in cooperative bird societies like the long-tailed tit, where all carers provision the brood significantly less in warmer days [63]. Hot temperatures may affect group members in different ways, like in the pied babbler *Turdoides bicolor*, where dominants but not helpers reduce their provisioning rate in hot days, with negative consequences on nestling growth [94]. Similarly, in chestnut-crowned babblers *Pomatostomus ruficeps,* the lack of rainfall, high wind speed and high temperature affect breeders and helpers differently and group coordination (measured as proportion of synchronous visits) declines in hot days [69].

However, a key question that remains unanswered is whether these short-term effects of weather conditions on provisioning behaviour translate into measurable consequences over the long term, given the current context of global warming. Here, we address this question in the cooperatively breeding carrion crow, by analysing an extensive data set, which covers the last twenty-six years.

In Spain, carrion crows form complex kin groups where up to seven subordinated individuals can join a dominant breeding couple [8]. Subordinates are non-breeding retained offspring or immigrants, mostly males, that are related to the same-sex breeder and that can sire chick in the brood [6]. Subordinate group members participate in brood care, boosting reproductive success [24], although their contribution can vary largely [22], with some birds refraining from providing care at all [5]. Recently, it has also been found that the degree of alternation of carer in provisioning the brood significantly improves the body condition of the nestlings, enhancing their post-fledging survival [90].

In this study, we first examine the effect of temperature and the number of days since the last rain on the provisioning rate of group members of different sex and social category (breeders/retained offspring/immigrants) and on the degree of alternation of carers in visiting the nest. Subsequently, we analyse how these two meteorological variables affect crow reproductive success, measured as the annual production of offspring and their body condition. We expect that, if high temperatures and lack of rain negatively affect both parental care and breeding success of crows, negative trends will emerge over the long term, due to global warming. To test this prediction, we analysed an extensive data set that encompasses the last 26 years of crow reproduction in a population living in the Northern Mediterranean region of Spain.

## Methods

### Study area and population

We studied a cooperatively breeding population of carrion crows in a 45Km^2^ area at La Sobarriba, Castilla y León, Northern Spain (42°37’, 5°26’W), characterized by a mosaic of crops, scrubs, oak forest patches, meadows, poplar and pine plantations and uncultivated land [7, 77]. Castilla y León region has a Mediterranean climate with long cold winters and short hot summers, being January the coldest month and July the warmest [4, 78]. In the last decades, monthly, seasonal and annual temperatures have increased in Spain [43, 75], especially during spring and summer [75], and rainfalls have decreased [76, 92]. Castilla y León, in particular, has changed toward a warmer and drier climate, notably in winter, spring and summer [36, 37].

In our study population, carrion crows form cohesive kin groups of up to nine individuals (average size ± SE = 3.2 ± 0.08; [5] that comprise a dominant breeding pair, its non-dispersing offspring, which can remain at natal territory for up to 4 years, and/or individuals, called “immigrants”, that fledged in other territories and settled in already established groups, where they are related to the resident breeder of the same sex [6]. Therefore, groups are extended families where subordinates can contribute to nestling care (nest building, feeding the incubating female and the chicks, and nest sanitation). Provisioning the brood is costly for crows, which lose mass in proportion to their effort [21, 23] and hence finely tune their contribution depending on extrinsic (food availability) and intrinsic (own condition) factors [5, 27]. Typically, breeders show the highest brood feeding rates, followed by male helpers and, lastly, the female helpers [22]. Helpers rise the total provisioning rate of the group, increasing nestling survival, and augment the probability of re-nesting after early nest failure [24]. A recent study also revealed that crow group members take turns in visiting the nest and that such coordination significantly increases the body condition of nestlings, boosting post-fledging survival rates [90].

### Data collection

Since 1995 we have monitored crow reproduction in the study area, by surveying all nests throughout the breeding season (March-July). Upon early nest failure, crows may renest up to three times in a season, but they never raise multiple successful broods [24]. Every year, nestlings were banded with colour rings and wind tags just before they left the nest (28–30 days old). Adult crows were captured with two-compartments walk-in traps and “snap traps” specifically developed for this species (for details on catching methods, see [8]. A sample of blood (200 µl) was taken from all the banded individuals for genetic analyses. The sex of each individual was determined by P2/P8 molecular method [49], while parentage analyses based on DNA microsatellites provided the breeding status of group members [9, 26].

We collected data on provisioning rate and timing of nest visits by placing camouflaged micro video cameras from a distance of ca. 1,5 – 2 m from nests with chicks older than 10 days [22] during the breeding seasons 1999, 2000, 2003–2007, 2015, 2018, 2019 and 2021. Daily recording bouts (lasting between 4 and 15 h) were distributed between 05:00 am and 08:00 p.m. (UTC time). Video recordings were analysed in the laboratory with VLC media player using slow motion when necessary, extracting the following data for each nest visit: the identity of the carer, the entrance and departure time from the nest (according to the UTC time displayed on all recordings) and the number of feeds delivered to chicks (i.e., the number of times the carer put its beak in a nestling’s open gape to regurgitate food [22]. We analysed a total of 2520 hours of video recordings that comprised 12,467 nest visits. We sampled 76 nests (average ± SE recording time per nest = 32 hours ± 3.56) in 50 different territories collecting data on 221 caregivers (68 breeding males, 69 breeding females, 45 helping male offspring, 11 helping male immigrants and 28 helping female offspring).

To analyse carers’ turn-taking, measured as the proportion of nest visits where a carer is followed by any other carer of the group) we restricted the data set to groups where all individuals were recognisable. The sample eventually comprised 58 nests from 40 different territories and 196 caregivers (64 breeding males, 63 breeding females, 31 helping male offspring, 11 helping male immigrants, 23 helping female offspring and 4 individuals of unknown sex and social category) for a total of 2157 hours of video recording (34.24 hours ± 4.29 per nest) and 11,309 nest visits.

Both in the analysis of feeding rate and carers’ alternation, six breeding males, six breeding females and two helping male offspring were sampled twice in different years, and one breeding male, two breeding females, one helping male immigrant, one helping male offspring and one helping female offspring three times. We retained them in the sample because, in all cases, they were observed in groups of different composition. In any case, their exclusion did not qualitatively change the results presented.

Reproductive success was measured as the number of chicks that survived until fledging. We collected data of 989 reproductive attempts in 125 territories during 1995–2021. Nestlings were measured when the eldest of the brood (hatching is asynchronous in crows, causing differences between 1 and 4 days among siblings; [24] was about to leave the nest, at the age of 28 – 30 days. Nestling body condition was quantified by dividing body mass by tarsus length, which is suitable lineal measure of structural size [60]. This index correlates with post-fledging survival in crows [90] and allows simplifying the statistical models, because it accounts for differences in body size due to sex and age. The sample comprised 901 chicks in 375 broods during the breeding seasons 1995–2021.

The Spanish National Agency of Meteorology (AEMET) provided data on hourly temperatures (in Celsius degrees) and daily rainfalls (mm) for the whole sampled period, collected at the weather station “Virgen del Camino”, located 11 km away from our study area.

### Statistical analyses

All data were analysed with Mixed Models with R 4.1.1. [73] with *lme4* package [10]. Normality of DARMHa scaled residuals, heteroscedasticity and outliers were checked with the package *DARMHa* [54], while multicollinearity between fixed factors was tested by computing variance inflation factors (VIFs) with the package *performance* [62]. The model significance test (Omnibus test) was performed by comparing the model of interest with the null model (without predictors) by means of Akaike’s Information Criterion values (AICc) [19], preserving the same structure of the random part. All models were fitted with random intercept fixed slopes, which always provided the best fit.

First, to test the effect of weather on crow provisioning rate, we built the data set by establishing three daytime periods, each comprising 5 hours (from 0500 to 1000 hours UTC; from 1001 to 1500, and from 1501 to 2000). Individual feedings rates were calculated as the number of feeds per hour delivered by a carer within a given daytime period. We used a Linear Mixed Model (LMM) fitted by Restricted Maximum Likelihood (REML) to analyse these data. The Box-Cox transformation [14] of the response term (frequency of feeds) improved the normality of the model residuals. The model included group size, chicks age, laying Julian date of the first egg of the clutch (taking first of march as reference), brood size, individual category, daytime period, average hourly air temperature for the corresponding daytime period, and number of days since the last rain as explanatory terms, as well as individual ID nested into group ID as random term to control for repeated measures. To detect possible differences among social categories of carers in their response to meteorology, we run a second model fitting two interactions, i.e., social category * temperature and social category *days since last rain. If significant, post hoc multiple comparisons across different categories of group members were performed with Package *Phia* using “Test Interactions” with X^2^ test and *p* values adjusted by Holm’s method [35].

Second, to investigate the influence of meteorological variables on carers’ turn-taking we used a Generalized Linear Mixed Model (GLMM), with binomial error distribution and logit link function, where two vectors comprising the number of alternate visits and repeated visits represented the response variable. This variable weights the proportion of alternated visits according to the total number of visits [32], which varies between social groups. This model included group size, chicks age, laying Julian date, brood size, daytime period, average hourly air temperature for each daytime period and number of days since the last rain as explanatory terms, and group ID as random term.

Third, to analyse the effect of temperature and lack of rain on nestling production we used a hurdle model, where the response variable is analysed in two steps. Initially, the model addresses the probability of attaining zero values, i.e. nest failure, and, subsequently, the probability of non-zero values (number of nestlings in successful broods). Hurdle models handle zero inflated distributions and are particularly suitable for analysing reproductive success in birds, where brood loss is generally caused by predation, while the number of nestlings in successful nests strongly depend on the quantity and quality of the care that they received [1, 64, 74]. The two steps procedure therefore allows telling apart the factors that influence each mechanism (predation/care) separately. For the present study, the second component is particularly relevant, as it directly relates with provisioning behaviour [24], and it will be the focus of our attention. The hurdle model here consists in a Generalized Linear Mixed Model using Template Model Builder (GLMMTMB) [16] with truncated Poisson distribution and log link function [12]. The number of nestlings was fitted as response variable. Group size, clutch size, laying Julian date, average daily maximum air temperature during the nestling development period (from hatching to fledging) and number of days without rain in that period represented the fixed terms, and group ID the random term (to control for different reproductive attempts in the same territory for a given year).

Fourth, to test whether meteorological variables affected the body condition of the chicks, we run a LMM fitted by REML, where group size, clutch size, laying Julian date, average daily maximum air temperature during the chick rearing period and number of days without rain during that period were fitted as explanatory variables, and brood ID as random term (to group nestlings from the same brood). The body condition index was Box-Cox transformed to improve the normality of the model residuals [14].

Furthermore, to model the change in nestling production and condition over the long-term, we used respectively: a GLMMTMB [16] with year as fixed term and group ID as random term, and a LMM fitted by REML with a Box-Cox transformation of the response variable, year as fixed term and brood ID as random term.

## Results

### Effect of weather on the provisioning rate

**Fig. 1:**
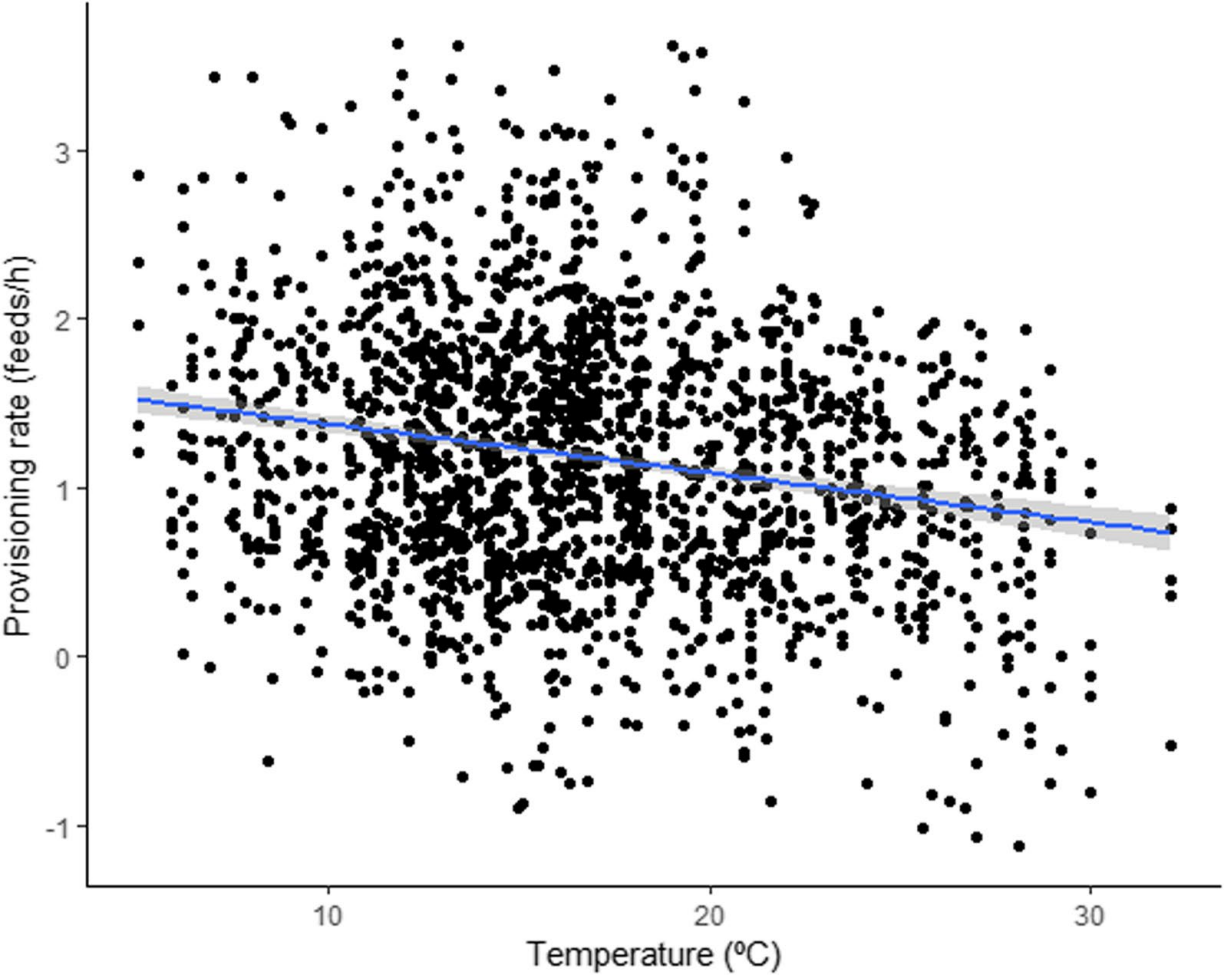
Fitted Box-Cox transformed values of provisioning rate (feeds/h) plotted against the average hourly air temperature (°C) for each daytime period. The shadowed area indicates 95% confidence limits.

**Fig. 2:**
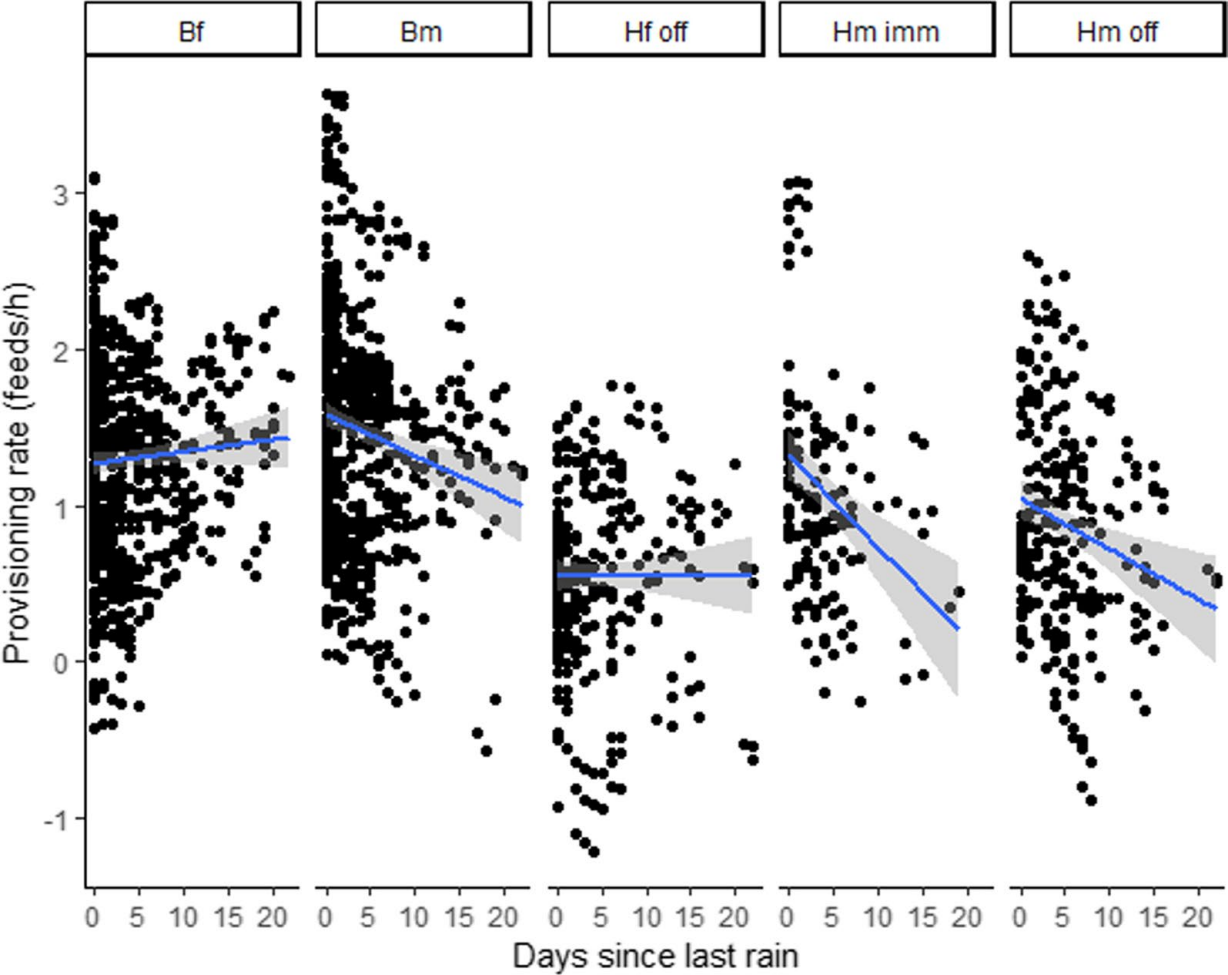
Fitted Box-Cox transformed values of provisioning rate (feeds/h) plotted against days since last rain for all categories of group members (Bm: breeder male, Bf: breeder female, Hf off: female offspring helper, Hm imm: male immigrant helper, Hm off: male offspring male). The shadowed areas indicate 95% confidence limits.

The provisioning rate of cares decreased significatively with rising temperatures (Table 1a, Fig. 1), regardless of the social category of carers (non-significant interaction category*temperature; Table 1b). Provisioning rate was also affected by the time elapsed since the last rain (Table 1a), but, in this case, the effect varied among categories of group members, as shown by the significant interaction category*days since last rain (Table 1b). In particular, the post-hoc analysis revealed significant differences between the breeding females, who slightly increased their contribution under dry conditions and the male helpers, both offspring and immigrant, who instead reduced their feeds (Additional file 1: Table S1, Fig. 2). As expected, based on previous results [22, 24], the provisioning rate differed among social categories, significantly decreased with group size and across daytime periods, and increased with brood size (Table 1a).

**Table 1:** Variables associated with individual provisioning rate.

Fixed terms	Estimate ± SE	df	t value	*p* value
*a*				
Group size	− 0.193 ± 0.057	83.889	− 3.388	**0.001**
Age chicks	− 0.010 ± 0.006	254.622	− 1.694	0.091
Laying Julian date	0.005 ± 0.004	76.229	1.343	0.183
Brood size	0.395 ± 0.030	518.381	13.066	**<0.001**
Category Bf	0.161 ± 0.077	141.361	2.097	0.038
Category Bm	0.360 ± 0.076	139.970	4.733	**<0.001**
Category Hf off	− 0.426 ± 0.105	173.808	− 4.049	**<0.001**
Category Hm imm	0.050 ± 0.153	159.563	0.328	0.743
Daytime 1	0.005 ± 0.030	1742.913	0.181	0.856
Daytime 2	0.111 ± 0.024	1713.039	4.599	**<0.001**
Temperature	− 0.024 ± 0.005	1744.409	− 5.290	**<0.001**
Days since rain	− 0.023 ± 0.006	1364.606	− 4.187	**<0.001**
*b*				
Temp:cat Bf	0.007 ± 0.006	1803.287	1.258	0.208
Temp:cat Bm	0.003 ± 0.006	1801.357	0.490	0.624
Temp:cat Hf off	− 0.006 ± 0.008	1782.818	− 0.800	0.424
Temp:cat Hm imm	− 0.008 ± 0.010	1769.339	− 0.813	0.416
Days since rain:cat Bf	0.028 ± 0.009	1539.082	3.256	**0.001**
Days since rain:cat Bm	0.002 ± 0.009	1462.369	0.187	0.852
Days since rain:cat Hf off	0.020 ± 0.013	1192.233	1.604	0.109
Days since rain:cat Hm imm	− 0.029 ± 0.014	1863.247	− 2.011	**0.044**

Results of Linear Mixed Models (LMMs) fitted by Restricted Maximum Likelihood (REML). *p* values obtained with Kenward-Roger’s method. Significant *p* values are highlighted in bold. a) Output of the model without interaction. b) output for the two interactions (temperature*category of carer and days since last rain*category of carer) added to model a. Bf: breeding female. Bm: breeding male. Hf off: Helper female offspring. Hm imm: Helper male immigrant. Hm off: helper male offspring (reference level for the variable *Category*). Day time 1: period from 0500 to 1000 hours UTC. Day time 2: period from 1000 to 1500 hours UTC. Day time 3: period from 1500 to 2000 hours UTC (reference level for the variable *Daytime*). Temperature refers to average hourly air temperature for the corresponding daytime period.

### Effect of weather on the degree of visit alternation

**Fig. 3:**
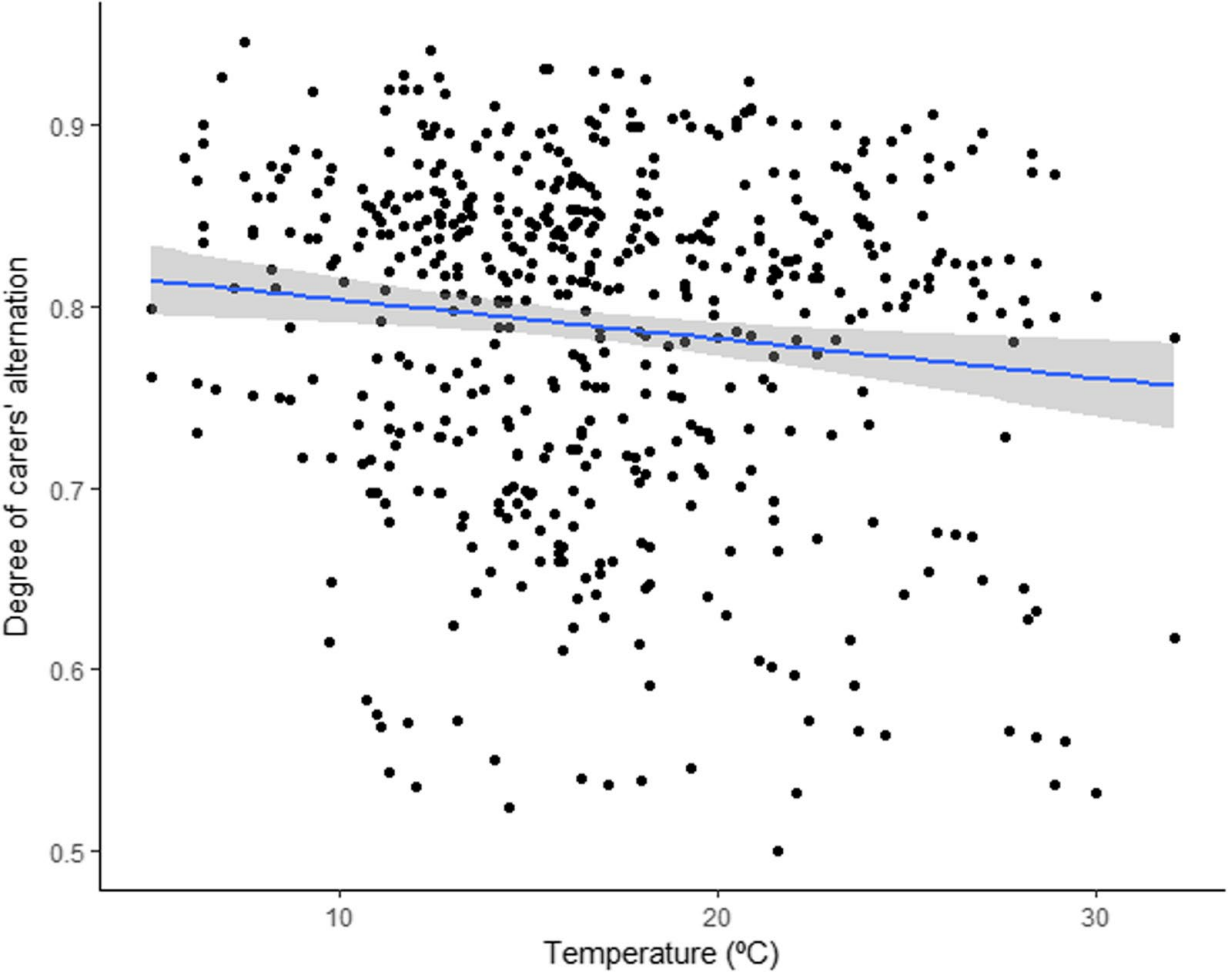
Degree of alternation of nest visits in relation to average hourly air temperature (°C) for each daytime period. Fitted values were plotted and the shadowed area indicates 95% confidence limits.

The degree of alternation of nest visits was negatively affected by rising temperature (Table 2, Fig.3). In contrast, the number of days since the last rain did not show a significant effect. Alternation also increased with group size, confirming previous results [90].

**Table 2:** Variables associated with alternation of nest visits

Fixed terms	Estimate ± SE	z value	*p* value
Group size	0.560 *±* 0.081	6.920	**< 0.001**
Age chicks	0.009 *±* 0.009	0.996	0.319
Laying Julian date	0.003 *±* 0.004	0.687	0.492
Brood size	0.059 *±* 0.040	1.483	0.139
Day time 1	0.063 *±* 0.049	1.279	0.201
Day time 2	0.018 *±* 0.040	0.456	0.648
Temperature	− 0.018 *±* 0.008	− 2.267	**0.023**
Days since rain	− 0.009 *±* 0.010	− 0.937	0.349

Results of Generalized linear mixed model (GLMM) with binomial error distribution fitted by maximum likelihood (Laplace Approximation). Significant *p* values are highlighted in bold. Temperature refers to average hourly air temperature for the corresponding daytime period.

### Effects of weather on nestling production and nestling body condition

The probability of nest failure significantly decreased with the size of the clutch (estimate ± SE = − 0.136 ± 0.067, Z = − 2.007, p = 0.045; Additional file 1^1^ Table S2a) and increased with the Julian laying date (estimate ± SE = 0.025 ± 0.009, Z = 2.709, p = 0.007) but did not depend on meteorological conditions. Similarly, the number of nestlings produced in successful nests was affected neither by temperature nor the number of days without rain, but increased with group size (estimate ± SE = 0.089 ± 0.027, Z = 3.239, p = 0.001; Additional file 1: Table S2b) and clutch size (estimate ± SE = 0.189 ± 0.04, Z = 4.718, p < 0.001), and decreased with the Julian laying date (estimate ± SE = − 0.014 ± 0.006, Z = − 2.543, p = 0.011), confirming previous results [24].

**Fig. 4:**
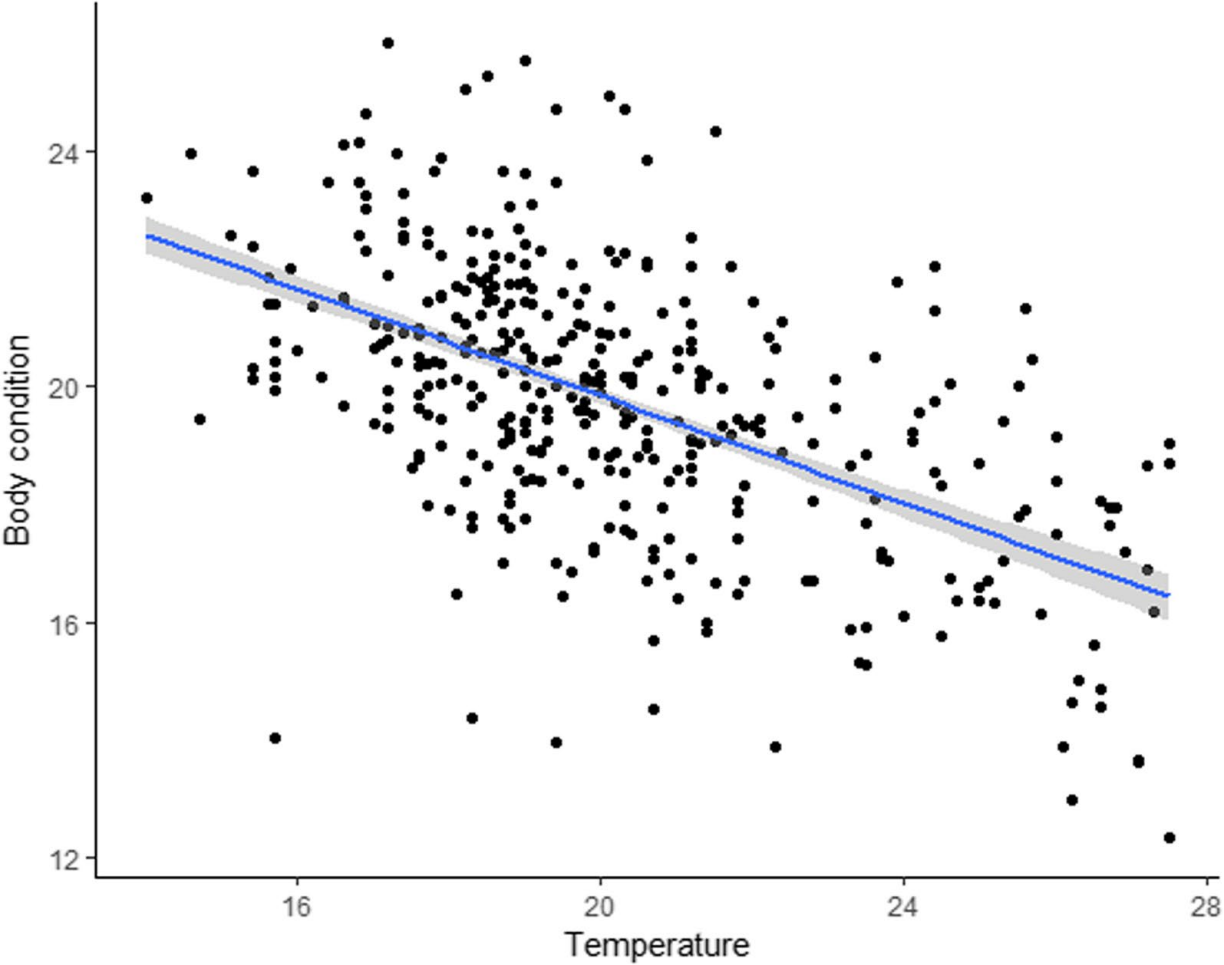
Effect of average daily maximum temperature during the chick rearing period on the nestling body condition. Fitted Box-Cox transformed values were plotted and the shadowed area indicates 95% confidence limits.

Average daily maximum air temperatures during the rearing period were negatively related to nestling body condition (Table 3, Fig. 4), while the number of days without rain showed no effect. No other variables proved significant in this analysis.

**Table 3:** Variables associated with nestling body condition

Fixed terms	Estimate ± SE	df	t value	*p* value
Group size	0.154 *±* 0.126	338.152	1.227	0.221
Clutch size	− 0.168 *±* 0.173	391.269	− 0.969	0.333
Laying Julian date	− 0.009 *±* 0.022	359.837	− 0.417	0.677
Temperature	− 0.361 *±* 0.118	368.027	− 3.072	**0.002**
Days without rain	− 0.065 *±* 0.046	356.789	− 1.418	0.157

Results of a LMM fitted by REML. *p* values obtained with Kenward-Roger’s method. Significant *p* values are highlighted in bold. The variable “Temperature” refers to average daily maximum air temperature during the chick rearing period. Days without rain were counted over the same period.

### Nestling production and condition trends over the long term.

**Fig. 5:**
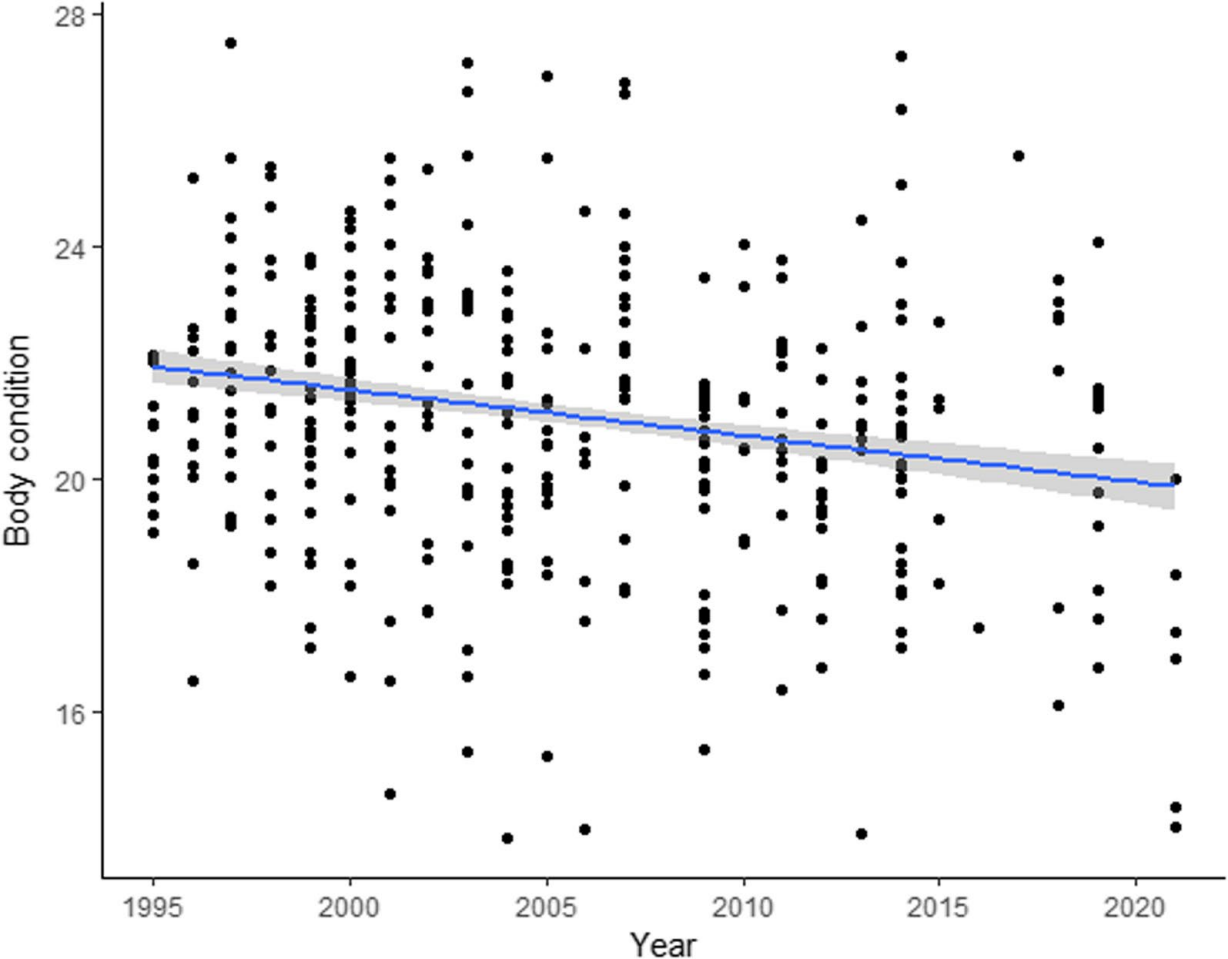
Variation of the body condition of chicks during the 26-years study period. Fitted Box-Cox transformed values were plotted and the shadowed area indicates 95% confidence limits.

The number of nestlings in successful nests did not vary over the 26-year study period (estimate ± SE= 0.001 ± 0.004, z value= 0.152, *p* value= 0.879). Nestling boy condition, instead, significantly worsened throughout the same period (estimate ± SE= − 0.067 ± 0.027, df= 361.295, t value= − 2.496, *p* value=0.013; Fig. 5).

## Discussion

Our results showed that high temperatures constrained crow provisioning behaviour and carers’ alternation suggesting a cascading effect on reproductive success. Lack of rain was also associated with a reduction in provisioning rate, particularly in helping males.

### Effect of temperature on provisioning behaviour and carers’ alternation

Increasing temperature negatively affected individual feeding rate, regardless of social category, and significantly reduced the degree of alternation of group members in provisioning the brood. High temperatures can reduce individual feeding rate in at least two non-exclusive ways: (1) by constraining the activity and mobility of invertebrates [42], which are the main preys of crows during the breeding period [31], (2) by forcing birds to lower their activity [86], spending more time near water supplies or shaded areas [45, 96] and increasing heat dissipation behaviour (wing spreading and panting behaviour, [39]. Indeed, we have frequently observed these behaviours in crows at high temperatures. Moreover, on days of intense hot, crow breeding females spend more time in the nest, shading the offspring.

Our data also showed that the degree of alternation at the nest of group members declines at high temperatures. Although the proximate mechanisms that allow crows to alternate nest visits are not yet fully understood [90], it seems likely that the need of resting in shaded areas, for example in tree canopies, might reduce the ability of crows to monitor each other behaviour and therefore to adjust own timing of nest visitation. In addition, stressful conditions that constraint individual provisioning rates and pose additional costs for thermoregulating might affect group members differently, for example depending on their current body condition, eventually disrupting the coordination of the group. Interestingly, a similar effect has been shown also in chestnut-crowned babblers, which normally synchronize their arrival at the nest to prevent brood predation, but, at high temperature, loose their coordination, particularly in large units [68, 69].

### Effect of lack of rain on provisioning behaviour and carers’ alternation

The effect of lack of rain on the provisioning rate was more complex than that of the temperature and significantly depended on the social category of the carer. Both offspring and immigrant male helpers showed the most substantial decreases, particularly compared with the breeding females, who instead slightly increased their effort under dry conditions.

Dry weather can cause a decrease in food availability [83] affecting foraging efficiency, but it may pose a less stringent physiological cost to crow activity, compared with high temperature. This may explain why some group members seem to cope with the lack of rain and can maintain (or even slightly increase) their feeding rates. A previous study, where food availability was experimentally manipulated, showed the helpers are more flexible in their investment in nestling care as compared with breeders, who maintained constant feeding rates [20] regardless on the current conditions. According to this, we found that male helpers, unlike breeders, responded to an increasing number of dry days by reducing their provisioning rate. Interestingly, however, female helpers maintained their effort under the same conditions. A recent study has shown that female helpers signal their contribution to brood provisioning to the dominant breeders and that their permanence in the territory depend on the perceived amount of help that they provide [89]. Assuming that the costs of lack of rain could be affordable for crows, female helpers might therefore be pressured to maintain their provisioning effort constant in order to retain group membership.

Unlike temperature, the time since the last rain showed no significant effect on the degree of carers’ alternation, indicating that, in spite of the reduced effort of some group members, carers might keep on monitoring each other behaviour and adjusting their own nest visit timing accordingly.

### Effect of weather on reproductive success

Meteorological stressors are known to affect nestling care in some biparental bird species, like the common fiscal *Lanius collaris* [34] and the southern yellow-billed Hornbill *Tockus leucomelas*. Complex effects have been reported in cooperatively breeding species that live in harsh environments, like the pied babbler, where breeders, unlike helpers, reduce their effort with increasing temperatures [94], and the chestnut-crowned babblers, where the social rank plays the opposite effect [69]. High temperatures also affect cooperative species that live at temperate latitudes, like the long-tailed tit [63], where all carers respond to heat by reducing their feeding rates.

Despite of the effect of weather on crow provisioning behaviour, the number of nestlings in successful nests did not significantly correlate with the temperature and the number of rain days during the nestling period. However, our data showed that the body condition worsened for nestlings that were raised during hot periods. This is consistent with the fact that high temperatures hinder carers’ alternation (this study) and hence the regularity of chick provisioning, which is known to affect the development of the nestlings [90]. High temperatures might also have influenced nestling growth more directly, generating a higher demand of energy and water for thermoregulation that carers might have not been able to satisfy [70].

In summary, crows proved sensitive to high temperature and lack of rain both at individual (food provisioning rate) and group level (alternation of nest visits), with immediate consequences on the body condition of their nestlings. Although caution should be taken given the correlative nature of our study, the results raise the question whether human induced global warming is already affecting crow reproduction, causing changes that may affect population persistence.

A recent meta-analysis [50] has revealed that climate change has been affecting avian offspring production only slightly at global scale, with migratory and larger-bodied species being more vulnerable than sedentary and smaller-bodied species. This suggest that the dramatic decline of many bird species around the globe [71, 79] reflects changes in adult/juvenile survival or a reduction of the proportion of the populations that breed [50]. Our data on carrion crows fit the former hypothesis. While offspring production did not vary significatively over the 26-years study period, we found a measurable negative trend in nestling body condition, which is, in this species, a key predictor of juvenile survival [25, 90]. Interestingly, crow populations are decreasing in Spain [2, 84], especially in the northern Mediterranean region, where our study population lives. Other corvid species are declining in the same area, particularly the jackdaw *Corvus monedula*, the magpie *Pica pica* and the raven *Corvus corax* [84], which instead has been reported to increase in desertic areas of North America [57]. These trends support the prediction that climate change will be especially adverse in the Mediterranean regions [48].

The effect of heat on nestling growth and conditions has been reported in several avian species [33, 34, 80, 85, 91], but long-term data are rarely available (see [13] for a notable exception). Our data on the carrion crows, strongly suggest a causal link between global warming and population dynamic, mediated through the effect of high temperature on brood caring behaviour and, ultimately, offspring conditions. More data are urgently needed to understand whether this could be a general process among birds.

## Supplementary Information

**Additional file 1. **Supplementary material.

## Data Availability

Analyses reported in this article can be reproduced using the data provided by Trapote et al., 2023 in Dryad.
