# Supplementary material for: Effects of meteorological conditions on brood care in cooperatively breeding carrion crow and consequences on reproductive success
Source: Front Zool. 2023 Jul 24;20:24. doi: 10.1186/s12983-023-00504-0 (PMC10364382; doi:10.1186/s12983-023-00504-0)
Supplement: Supplementary file 1 — Additional file 1. Supplementary material. [file 12983_2023_504_MOESM1_ESM.docx]

**SUPPLEMENTARY MATERIAL**

**Table S1**. Post-hoc analysis of the interaction category * days since rain (X^2^ Test, P-value adjusted by Holm’s method). Bm: breeder male, Bf: breeder female, Hf off: female offspring helper, Hm imm: male immigrant helper, Hm off: male offspring male. Significant p values are highlighted in bold.

| *Pairwise comparisons* | *Value* | *Df* | *X^2^* | *Pr (>X^2^)* |
| --- | --- | --- | --- | --- |
| *Bf-Bm* | 0.026 | 1 | 4.686 | 0.218 |
| *Bf-Hf off* | 0.008 | 1 | 0.236 | 1.000 |
| *Bf-Hm imm* | 0.057 | 1 | 9.195 | **0.024** |
| *Bf-Hm off* | 0.049 | 1 | 9.049 | **0.024** |
| *Bm-Hf off* | -0.018 | 1 | 1.192 | 0.825 |
| *Bm-Hm imm* | 0.031 | 1 | 2.547 | 0.552 |
| *Bm-Hm off* | 0.023 | 1 | 1.840 | 0.7 |
| *Hf off-Hm imm* | 0.049 | 1 | 4.872 | 0.218 |
| *Hf off-Hm off* | 0.041 | 1 | 4.239 | 0.237 |
| *Hm imm-Hm off* | -0.008 | 1 | 0.128 | 1.000 |

**Table S2.** Variables associated with the probability of nest failure (a) and the number of nestlings produced in successful nests (b). Results of a Hurdle model. P values obtained with Kenward-Roger’s method. Significant p values are highlighted in bold. The variable “Temperature” refers to average daily maximum air temperature during the chick rearing period. Days without rain were counted over the same period.

|  | *Estimate ± SE* | *Z value* | *Pr (>\|z\|)* | |
| --- | --- | --- | --- | --- |
| ***a. Zero-inflation model*** |  |  |  | |
| *Group size* | -0.079 ± 0.056 | -1.415 | 0.157 | |
| *Clutch size* | -0.136 ± 0.067 | -2.007 | **0.045** | |
| *Temperature* | -0.021 ± 0.048 | -0.443 | 0.657 | |
| *Days without rain* | -0.024 ± 0.02 | -1.213 | 0.225 | |
| *Laying Julian date* | 0.025 ± 0.009 | 2.709 | **0.007** |  |
| ***b. Conditional model*** |  |  |  | |
| *Group size* | 0.089 ± 0.027 | 3.239 | **0.001** | |
| *Clutch size* | 0.189 ± 0.04 | 4.718 | **< 0.001** | |
| *Temperature* | 0.02 ± 0.028 | 0.701 | 0.483 | |
| *Days without rain* | -0.014 ± 0.011 | -1.278 | 0.20 | |
| *Laying Julian date* | -0.014 ± 0.006 | -2.543 | **0.011** | |
